# Ayahuasca-assisted meaning reconstruction therapy as an early resource for bereavement: a non-randomized clinical trial

**DOI:** 10.1038/s41598-025-13251-5

**Published:** 2025-09-01

**Authors:** Oscar Soto-Angona, Oscar Andión, Pablo Sabucedo, Robert A. Neimeyer, Josep Maria Haro, Julia Javkin, Magí Farré, Débora González

**Affiliations:** 1 Department of Psychiatry and Forensic Medicine, Universitat Autonoma de Barcelona, Cerdanyola del Vallés, Spain; 2 Sociedad Española de Medicina Psicodélica (SEMPsi), Barcelona, Spain; 3 Parc Sanitari Sant Joan de Déu, Sant Boi de Llobregat, Spain; 4 Institut de Recerca Sant Joan de Déu (IRSJD), Esplugues de Llobregat, Spain; 5 Research Sherpas, Girona, Spain; 6 Faculty of Health and Life Sciences, University of Liverpool, Liverpool, UK; 7 Portland Institute for Loss and Transition, Portland, OR USA; 8 Center for Biomedical Research in Mental Health (CIBERSAM), Madrid, Spain; 9 Kiyumi Collective, Hoosfdorp, Netherlands; 10 Heart & Brain Training, Nijmegen, Netherlands; 11 PHI Association, Barcelona, Spain; 12 Department of Clinical Pharmacology, Hospital Universitari Germans Trias I Pujol-IGTP, Badalona, Spain; 13 Departament of Pharmacology, Therapeutics and Toxicology, Universitat Autonoma de Barcelona, Cerdanyola del Vallés, Spain; 14 Faculty of Health Sciences, Isabel I University, Burgos, Spain; 15 Clinica Synáptica, Barcelona, Spain

**Keywords:** Ayahuasca, Psychedelic-assisted therapy, Meaning reconstruction, Grief, Bereavement, Prolonged grief disorder, Clinical trial, Diseases, Health care

## Abstract

Preliminary evidence suggests that ayahuasca may alleviate severe grief symptoms. This three-arm, sequentially allocated, open-label study examines the therapeutic changes associated with ayahuasca-assisted meaning reconstruction therapy (A-MR) compared to meaning reconstruction therapy alone (MR) and a no-treatment control (NT). A total of 84 adults experiencing severe grief within 12 months of losing a first-degree relative were allocated to A-MR (*n* = 28), MR (*n* = 28), or NT (*n* = 28). Grief severity, prolonged grief disorder symptoms, post-traumatic growth, and quality of life were assessed at baseline, after the intervention, and 3 months post-intervention. Ayahuasca was well tolerated, with no serious adverse events reported. All groups showed significant grief severity reduction (A-MR: *p* < .0001, *d* = 2.44; MR: *p* < .0001, *d* = 1.84; NT: *p* < .002, *d* = 0.74). Greater reductions were observed in the A-MR compared to MR (*p* = .012, *d* = 0.86) and NT (*p* = .0008, *d* = 1.07). A-MR was also associated with significant improvements in prolonged grief symptomatology, post-traumatic growth, and quality of life, with medium-to-large effect sizes. This is the first controlled prospective study to provide preliminary support for A-MR as a safe and potentially effective intervention for severe grief, though replication in larger randomized trials is required.

**Supplementary Information:**

The online version contains supplementary material available at 10.1038/s41598-025-13251-5.

## Introduction

The word “ayahuasca”, meaning “vine of the soul” in Quechua, refers to a concoction that has been used by various Amazonian cultures for spiritual, communal, and healing purposes since pre-Columbian times^1^. Ayahuasca is a natural plant-based brew typically composed of the bark of the *Banisteriopsis caapi* vine and the leaves of the *Psychotria viridis* shrub^2^. The main psychoactive compounds in the bark are monoamine oxidase inhibitors (MAOIs), which enable N, N-dimethyltryptamine (N, N-DMT), present in the leaves of the shrub, to exert its effects on the central nervous system^3^. N, N-DMT is a tryptamine that acts primarily as an agonist of the 5-HT2A receptor, but also exhibits activity at trace amine-associated receptors (TAARs) and sigma-1 receptors, interacting with the serotonin, glutamate, dopamine, and acetylcholine systems^4^.

Animal, in vitro, and human studies have shown that ayahuasca, like other serotonergic psychedelics, stimulates adult neurogenesis and neuroplasticity^5–7^ within a temporal window ranging from a few hours after administration to several weeks^8^. Post-acute effects include enhanced mindfulness capacities^9^creative divergent thinking^10^psychological flexibility^11^and cognitive reappraisal^12^which may promote new behavioral patterns if taking place as part of a psychotherapeutic process. A single dose of ayahuasca has been shown to reduce depressive symptoms and suicidality, with effects lasting from 7^13^ to 21 days^14^. The subjective perceptual effects evoked by ayahuasca have been correlated with reductions in depressive symptoms, underscoring the therapeutic importance of the phenomenological experience itself^13^. In addition to depression, preliminary evidence also indicates that ayahuasca may have the potential to alleviate grief symptoms after the loss of a loved one, although further research is needed^15,16^. Qualitative data suggest that ayahuasca may confer therapeutic benefits in the context of grief by eliciting subjective experiences that facilitate emotional processing, meaning-making of past events, and the reconstruction of identity and existential meaning^16^. Moreover, ayahuasca may facilitate the continuation of the bond with the deceased by evoking a new positive internal representation of them during its acute effects, thereby contributing to the redefinition of attachment to the deceased^17^.

Grief, even when involving profound emotional turmoil and preoccupation with the deceased, is a normal and unavoidable aspect of human life. Most bereaved people experience moderate-to-high levels of grief shortly after the loss, often leading to disengagement from life and prior activities^18^. Although distress and avoidance normally decrease over time, a significant proportion of the population experiences different grief trajectories^19^. The fact that clinical research and intervention often focus on prolonged and functionally impairing grief, beyond 6 to 12 months after the loss, does not negate that the consequences of complex grief can be present earlier. The death of a close relative, for example, has been linked to an increased risk of suicide, self-harm, and psychiatric illness within the first year of the loss^20–22^. A decline in physical health, particularly cardiovascular health^23,24^has also been associated with recent bereavement, contributing to a heightened mortality risk within the first six-month period after spousal loss^25^.

To date, meta-analyses have not found evidence supporting the effectiveness of any early psychological intervention in preventing Prolonged Grief Disorder (PGD)^26,27^. Additionally, there is no evidence that antidepressant medication is effective as a stand-alone treatment for PGD^28^. However, several studies have identified a notable increase in antidepressant prescriptions during the second month after bereavement, which tends to decline over the following year^29,30^. The development of effective and preventative early grief support therapies, therefore, could help decrease the risk of PGD, while also mitigating the early physical and emotional consequences for those who are most vulnerable.

This study aims to investigate the efficacy of an ayahuasca-assisted therapy based on a meaning reconstruction approach^31^focusing on bereaved people experiencing severe grief symptoms within the first year after the death of a loved one. Our primary hypothesis is that this intervention will lead to a significant reduction in grief symptom severity and PGD symptomatology, compared to the meaning reconstruction therapy alone and a no-intervention control group. In addition to this, we also hypothesize an increase in both post-traumatic growth and quality of life in the experimental group. We expect these changes to be sustained over a 3-month follow-up period.

## Results

### Participants and attrition

**Fig. 1:**
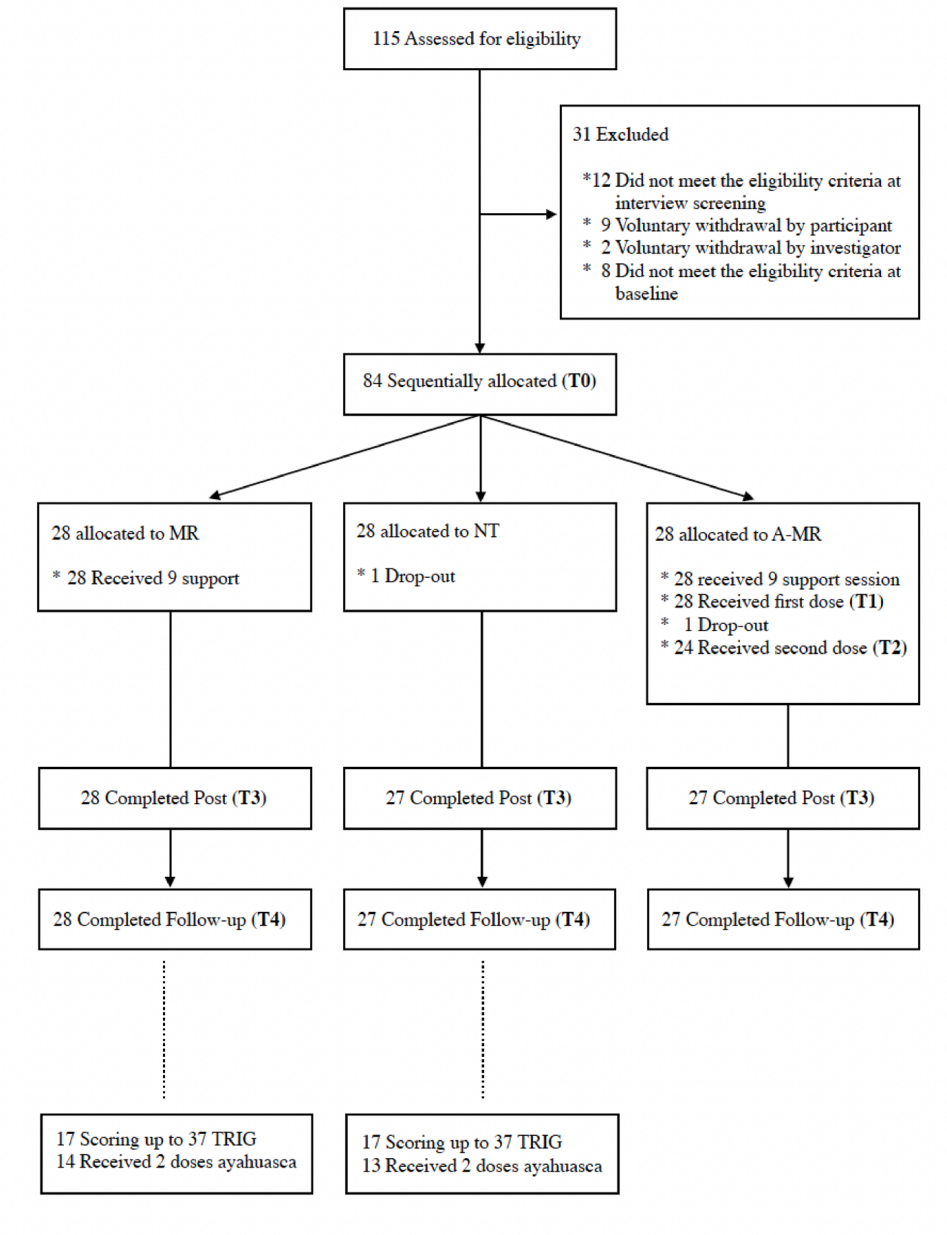
Flowchart of participants.

A total of 84 participants were allocated sequentially to the three study groups (Fig. 1).

A single participant dropped out of the experimental Ayahuasca-assisted Meaning Reconstruction therapy group (A-MR) following the first ayahuasca session, and a second participant in the No-Treatment group (NT) declined further participation before the post-intervention assessments (T3 and T4). Participants who dropped out had higher baseline grief scores compared to the mean score of those who completed the study (TRIG (T0): 63 and 60 vs. 52.4(7.1); t_(83)_ ≥ 9.86; *ps* ≤ 0.001; and TGI-SR (T0): 74 and 77 vs. 60.86 (12.2); t_(83)_ ≥ 9.87; *ps* ≤ 0.001). No participant dropped out of the Meaning Reconstruction group (MR). Total dropouts accounted for 2.4% of the total sample.

A total of fifty-five participants completed the 9-session-long psychotherapeutic component of the treatment (A-MR group: *n* = 27; MR group: *n* = 28). A total of 27 participants completed the first ayahuasca session, and 24 completed the second ayahuasca session in the A-MR group. No differences in any of the primary or secondary outcomes studied were observed when A-MR group participants who attended one or two ayahuasca sessions were compared (T0: *ps* ≥ 0.194; T3: *ps* ≥ 0.353; and T4: *ps* ≥ 0.538).

A low frequency of missing values (*n* = 15; 0.40%) was observed. Moreover, missing values were observed only in 6 participants. Little’s MCAR test (c^2^_(72)_ distance = 76.8; *p* = .328) and a logistic regression analysis were conducted to evaluate missing data, showing no relationships with the variables included in the study (all *ps* > 0.999).

The baseline characteristics of the total sample are presented in Table 1. No significant between-group differences were observed in any of the descriptive variables (*ps* ≥ 0.085).

**Table 1:** Baseline sociodemographic characteristics of participants (total sample and by group).

	Total sample	A-MR	MR	NT	*p*
	*n* = 84 (%)	*n* = 28 (%)	*n* = 28 (%)	*n* = 28 (%)	
Age, mean (SD)	43.2 (11.7)	43.8 (10.8)	44.6 (12.4)	41.1 (11.9)	0.497
Months since death, mean (SD)	5.9 (3.4)	5.8 (3.7)	6.7 (3.1)	5.2 (3.3)	0.218
Female	54 (64.3)	19 (67.8)	18 (64.3)	17 (63.0)	0.856
Marital status					
Single	26 (30.1)	6 (21.4)	8 (28.6)	12 (42.8)	0.447
Married/stable partner	41 (48.8)	16 (57.1)	13 (46.4)	12 (42.8)	
Divorced	11 (13.1)	5 (17.9)	3 (10.7)	3 (10.7)	
Widowed	6 (7.1)	1 (3.6)	4 (14.3)	1 (3.6)	
With university studies	70 (83.3)	24 (85.7)	23 (82.1)	23 (82.1)	0.999
Caucasian	75 (89.3)	26 (92.8)	27 (96.4)	22 (78.6)	0.134
Religious	6 (7.1)	1 (3.6)	2 (7.1)	3 (10.7)	0.867
Relationship of the deceased					
Parent	50 (59.5)	20 (71.4)	15 (53.6)	15 (53.6)	0.389
Partner	8 (9.5)	1 (3.6)	5 (17.8)	2 (7.1)	
Son/daughter	15 (17.9)	4 (14.3)	6 (21.14)	5 (17.8)	
Brother/sister	11 (13.1)	3 (10.7)	2 (7.1)	6 (21.4)	
Cause of death					
Disease	46 (54.8)	19 (67.8)	16 (57.1)	11 (39.3)	0.269
Aging	10 (11.9)	4 (14.3)	3 (10.7)	3 (10.7)	
Suicide	4 (4.8)	1 (3.6)	2 (7.1)	1 (3.6)	
Accident	16 (19.0)	2 (7.1)	6 (21.4)	8 (28.6)	
Other/unknown	8 (9.5)	2 (7.1)	1 (3.6)	5 (17.8)	
Previous medication	8 (9.5)	3 (10.7)	4 (14.3)	1 (3.6)	0.520
Previous psychotherapy	22 (26.3)	8 (28.6)	7 (25.0)	7 (25.0)	0.940
Previous psychedelic use	62 (73.8)	25 (89.3)	18 (64.3)	19 (67.8)	0.085
Previous ayahuasca use	29 (34.5)	7 (25.0)	11 (39.3)	11 (39.3)	0.431

### Safety

The acute effects of ayahuasca were well tolerated by all participants of the A-MR group, with no serious adverse events reported. The participant who declined further participation after the first ayahuasca experience claimed to see no meaning to ayahuasca sessions. Four participants did not take the second dose of ayahuasca, alleging not feeling psychologically prepared for another session so soon after the first one (*n* = 1), or due to a personal commitment on the day of the second administration (*n* = 3). Eight reported experiencing a negative effect during the ayahuasca session, and three were uncertain. Reported effects included nausea, vomiting or diarrhea (*n* = 16), headache (*n* = 4), fainting (*n* = 2), and anguish (*n* = 2) during the ayahuasca session. Only 3 participants perceived an adverse event the week following the ayahuasca session, including headache (*n* = 1), distraction, (*n* = 1) and minor sickness (*n* = 1). Open answers are shown in **Appendix 1.**

The average of the total PANSS-EC score was 5.07 (0.267) at 24 h post-administration. No participants required psychiatric medication within the 24-hour period following administration. Only two participants in the experimental group (A-MR) required Ibuprofen 8 h post-administration due to persistent headache.

### Level of satisfaction with the support program and ayahuasca sessions

From the A-MR and MR groups Total sample (*n* = 55), 78.2% were very satisfied with the support program [A-MR: *n* = 20; MR: *n* = 23], 18.2% somewhat satisfied [A-MR: *n* = 6; MR: *n* = 4], 1.8% somewhat dissatisfied [A-MR: *n* = 1], and 1.8% were very dissatisfied [MR: *n* = 1]. Regarding the ayahuasca sessions in the A-MR group, 81.5% were very satisfied, 11.1% were somewhat satisfied, 3.7% were somewhat dissatisfied, and 3.7% were very dissatisfied. Open answers are shown in **Appendix 2.**

### Primary outcomes

The descriptive statistics for the primary outcomes are reported in Table 2. For both measures studied, an *Intervention x Time* interaction was observed (TRIG: F_(3.57; 139.13)_ = 22.70; *p* < .0001; η*p*^2^ = 0.37; TGI-SR: F_(3.57; 139.37)_ = 24.88; *p* < .0001; η*p*^*2*^ = 0.39). However, no interaction effect of the covariate ‘Months Since Death’ was observed in the TRIG analysis (F_(1.78; 139.13)_ = 2.47; *p* = .094; η*p*^2^ = 0.03 and F_(1; 78)_ = 1.06; *p* = .307; η*p*^2^ = 0.01; respectively) or in the TGI-SR analyses (F_(1.79; 139.37)_ = 1.25; *p* = .286; η*p*^2^ = 0.02 and F_(1; 78)_ = 0.55; *p* = .461; η*p*^2^ < 0.01; respectively).

The analysis of the interactions observed for the severity of grief symptoms (TRIG) did not show differences between groups at baseline (T0: *ps* > 0.999), nor between the MR and the NT groups at post-intervention or at the 3-month follow-up (T3 and T4: *ps* ≥ 0.961). However, a higher reduction in normal grief severity was observed in the A-MR group compared to the NT group [T3: *p* < .0001; Cohen’s *d* = 1.50; T4: *p* < .0001; Cohen’s *d* = 1.25] and with the MR group [T3: *p* = .0001; Cohen’s *d* = 1.20; T4: *p* = .0008; Cohen’s *d* = 1.05] at both post-intervention measures (Table 3).

**Fig. 2:**
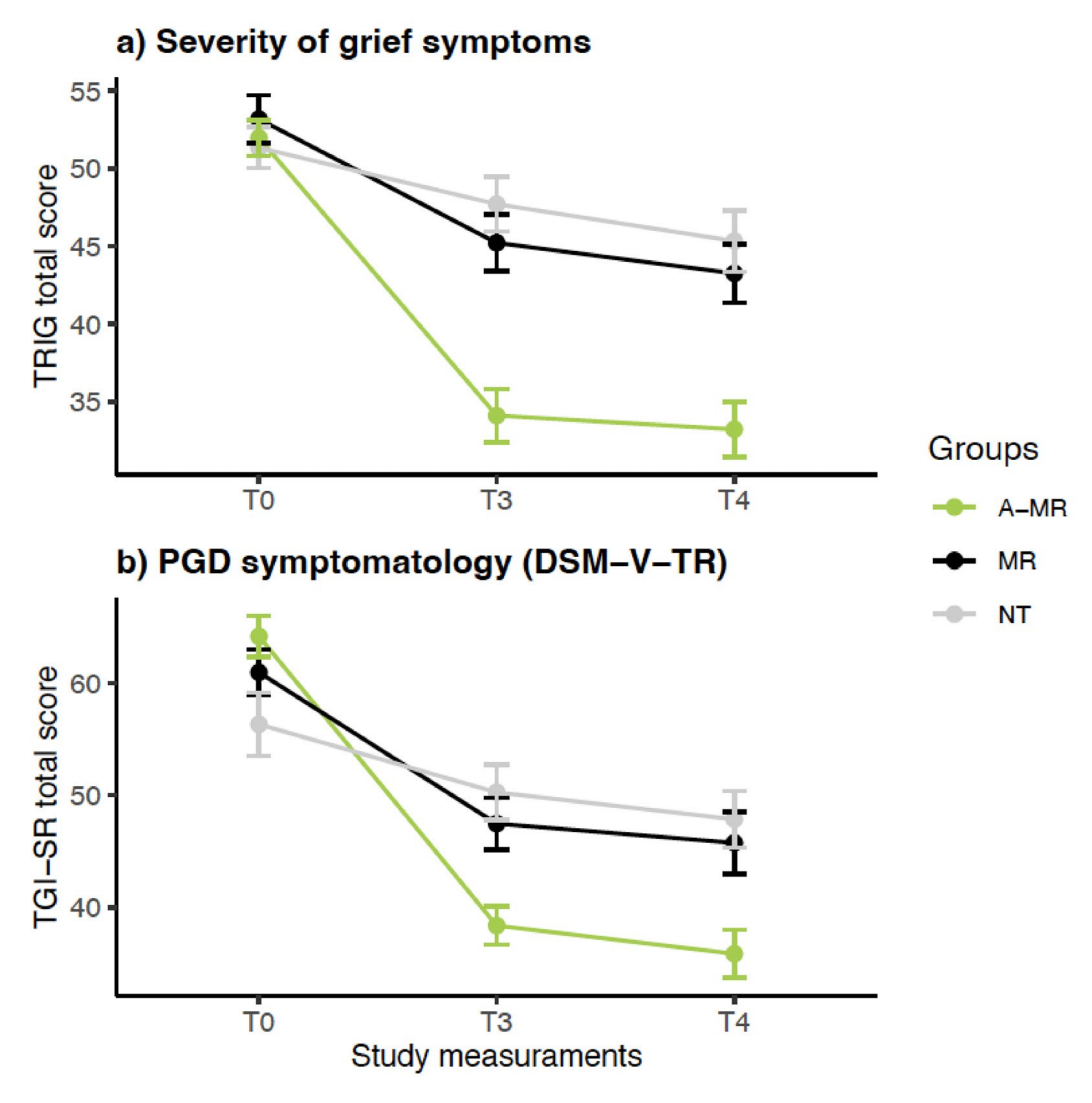
Changes in primary outcomes at different time points. A-MR: Ayahuasca-assisted Meaning Reconstruction group; MR group: Meaning Reconstruction group; NT group: No treatment group; T0: Baseline; T3: Post-treatment; T4: 3 Months follow-up. TRIG: Texas Revised Inventory of Grief; TGI-SR: Traumatic Grief Inventory-Self Report; PGD: Prolonged Grief Disorder; DMS-V-TR: Diagnostic and Statistical Manual of Mental Disorders, Fifth Edition, Text Revision.

The within-group comparison of TRIG showed a reduction in grief severity for all groups at post-intervention [A-MR (T0 vs. T3): *p* < .0001; Cohen’s *d* = 2.13; MR (T0 vs. T3): *p* < .0001; Cohen’s *d* = 1.48; NT (T0 vs. T3): *p* = .02; Cohen’s *d* = 0.59] and at the follow-up assessment [A-MR (T0 vs. T4): *p* < .0001; Cohen’s *d* = 2.46; MR(T0 vs. T4): *p* < .0001; Cohen’s *d* = 1.86; NT (T0 vs. T4): *p* = .004; Cohen’s *d* = 0.86]. No reductions were observed between T3 and T4 for any of the groups (*ps* ≥ 0.156) (Fig. 2a).

The analysis of the *Intervention x Time* interaction for PGD symptoms (TGI-SR) showed no differences between groups at baseline (T0: *ps* ≥ 0.06) or between the MR and NT groups at post-intervention or follow-up (T3 and T4: *ps* ≥ 0.990).

However, differences were observed at both post-intervention assessment points between the A-MR and the MR groups [T3: *p* = .012; Cohen’s *d* = 0.86; T4: *p* = .018; Cohen’s *d* = 0.76], and between the A-MR and the NT groups [T3: *p* = .0008; Cohen’s *d* = 1.07; T4: *p* = .004; Cohen’s *d* = 0.99] (Table 3).

Within-group comparisons of TGI-SR showed reductions in all 3 groups at post-intervention [A-MR (T3 vs.T0): *p* < .0001; Cohen’s *d* = 2.44; MR (T3 vs.T0): *p* < .0001; Cohen’s *d* = 1.84; NT (T3 vs.T0): *p* = .002; Cohen’s *d* = 0.74] and at follow-up [A-MR (T4 vs.T0): *p* < .0001; Cohen’s *d* = 2.48; MR (T4 vs.T0): *p* < .0001; Cohen’s *d* = 1.81; NT (T4 vs.T0): *p* = .0001; Cohen’s *d* = 0.96]. No reductions were observed between T3 and T4 for any of the groups (*ps* ≥ 0.196) (Fig. 2b).

### Secondary outcomes

The descriptive statistics for the secondary outcomes are reported in Table 2. The RM-ANCOVA performed to test the effect of treatment on post-traumatic growth (PTSG-SF) showed an interaction of *Intervention* x *Time* (F_(3.55; 138.26)_ = 9.51; *p* < .0001; h*p*^*2*^ = .20). However, no interaction effect of the covariate ‘Months Since Death’ was observed (F_(1.77; 138.26)_ = 0.750; *p* = .474; h*p*^2^ = 0.01 and F_(1; 78)_ = 0.062; *p* = .804; h*p*^2^ < 0.01; respectively).

**Fig. 3:**
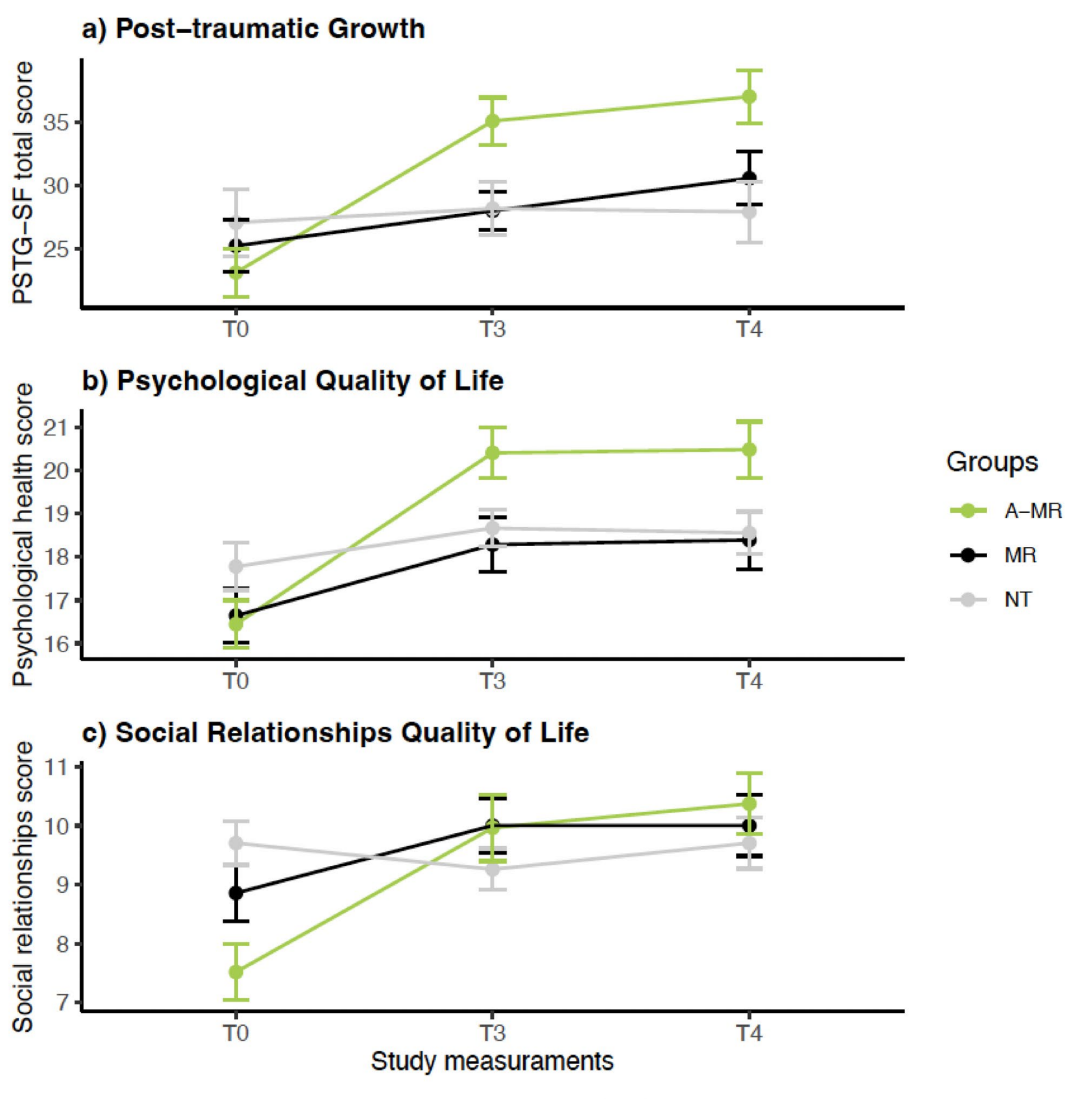
Changes in secondary outcomes at different time points. A-MR: Ayahuasca-assisted Meaning Reconstruction group; MR group: Meaning Reconstruction group; NT group: No treatment group; T0: Baseline; T3: Post-treatment; T4: 3 Months follow-up; PTSG-SF: Posttraumatic Growth Inventory‐Short Form; Psychological health: Psychological health domain of the World Health Organization Quality of Life (WHOQOL-BREF); Social relationships: Social relationships domain of the World Health Organization Quality of Life (WHOQOL-BREF).

The analysis of the interaction showed no significant differences between groups in PTSG-SF at baseline (T0: *p*s ≥ 0.650). In addition, no significant differences were observed between the MR and the NT groups at post-intervention or follow-up (T3 and T4: *ps* ≥ 0.998). However, higher PTSG-SF scores were observed for the A-MR group compared to the MR group at post-intervention [T3: *p* = .023; Cohen’s *d* = 0.80], as well as compared to the NT group at post-intervention [T3: *p* = .029; Cohen’s *d* = 0.67] and follow up [T4: *p* = .014; Cohen’s *d* = 0.77] (see Table 2 and Fig. 3).

**Table 2:** Descriptive statistics for the primary and secondary outcomes at baseline (T0), post-intervention (T3) and 3-month follow-up (T4). Values are given as mean differences (with standard deviations).

	A-MR (*n* = 27)			MR (*n* = 28)			NT (*n* = 27)		
	T0	T3	T4	T0	T3	T4	T0	T3	T4
	Mean (SD) *	Mean (SD)	Mean (SD)	Mean (SD)	Mean (SD)	Mean (SD)	Mean (SD)*	Mean (SD)	Mean (SD)
TRIG	52.0 (5.91)	34.1 (8.90)	33.2 (9.18)	53.2 (8.12)	45.2 (9.59)	43.2 (9.92)	51.3 (6.90)	47.7 (9.18)	45.0 (10.20)
TGI-SR	64.2 (9.43)	38.4 (8.86)	35.9 (11.10)	61.0 (10.78)	47.5 (12.14)	45.8 (14.62)	56.3 (14.72)	50.3 (12.95)	47.2 (13.15)
PSGI-SF	23.1 (9.82)	35.1 (9.62)	37.0 (10.92)	25.2 (10.82)	28.0 (8.01)	30.6 (11.05)	27.1 (13.77)	28.2 (10.96)	27.9 (12.44)
QoL-P. H	16.4 (2.89)	20.4 (3.09)	20.5 (3.37)	16.6 (3.34)	18.3 (3.35)	18.4 (3.54)	17.8 (2.82)	18.7 (2.22)	18.6 (2.56)
QoL-S. R	7.52 (2.46)	9.96 (2.93)	10.37 (2.68)	8.86 (2.49)	10.0 (2.45)	10.0 (2.74)	9.70 (1.92)	9.26 (9.26)	9.70 (2.25)

A-MR: Ayahuasca-assisted Meaning Reconstruction group; MR group: Meaning Reconstruction group; NT group: No treatment group. TRIG: Texas Revised Inventory of Grief total score; TGI-SR: Traumatic Grief Inventory Self-Report total scores; PSGI-SF: Post-traumatic Growth Inventory-Short Form total score; QoL-P.H: Psychological Health domain of the World Health Organization Quality of Life (WHOQOL-BREF); QoL-S. R: Social Relationships domain of the World Health Organization Quality of Life (WHOQOL-BREF).

**Table 3:** Between-group and within-group effect sizes [Cohen’s d (95% CI)] for the primary and secondary outcome variables.

	Between-groups							Within-group					
	A-MR vs. MR		A-MR vs. NT		MR vs. NT			A-MR		MR		NT	
	d	95%CI	d	95%CI	d	95%CI		d	95%CI	d	95%CI	d	95%CI
TRIG **(T0)**	0.17	0.16-0.19	0.10	0.08-0.13	0.24	0.23–27							
TRIG **(T3)**	1.20***	1.19–1.23	1.50****	1.51–1.55	0.27	0.26-0.30	TRIG **(T3** vs. **T0)**	2.13****	2.17–2.22	1.48****	1.48–1.52	0.59*	0.59-0.61
TRIG **(T4)**	1.05***	1.05–1.09	1.25***	1.26–1.29	0.21	0.19-0.23	TRIG **(T4** vs. **T0)**	2.46****	2.51–2.56	1.86****	1.90–1.95	0.86**	0.85-0.87
TGI-SR **(T0)**	0.32	0.31-0.34	0.64	0.63-0.67	0.36	0.36–39							
TGI-SR **(T3)**	0.86*	0.85-0.89	1.07**	1.07–1.11	0.22	0.20-0.24	TGI-SR **(T3** vs. **T0)**	2.44****	2.47–2.52	1.84****	1.87–1.91	0.74*	0.74-0.77
TGI-SR **(T4)**	0.76*	0.77–81	0.99**	0.98-1.02	0.15	0.14-0.17	TGI-SR **(T4** vs. **T0)**	2.48****	2.51–2.56	1.81****	1.86–1.89	0.96***	0.96-0.98
PTSG-SF **(T0)**	0.20	0.20-0.24	0.33	0.32-0.35	0.15	0.13-0.17							
PTSG-SF **(T3)**	0.80*	0.79-0.83	0.67*	0.66-0.70	0.02	0.003-0.04	PTSG-SF **(T3** vs. **T0)**	1.30****	1.31–1.33	0.28	0.28-0.31	0.10	0.09-0.11
PTSG-SF **(T4)**	0.59	0.57-0.61	0.77*	0.77–81	0.22	0.22-0.25	PTSG-SF **(T4** vs. **T0)**	1.78****	1.80–1.84	0.57*	0.58-0.61	0.09	0.08-0.10
QoL-P.H **(T0)**	0.06	0.04-0.08	0.47	0.45-0.50	0.36	0.36.-39							
QoL-P.H **(T3)**	0.66*	0.65-0.68	0.65	0.64-0.68	0.13	0.12-0.15	QoL-P.H **(T3** vs. **T0)**	1.18****	1.18–1.21	0.63**	0.62-0.64	0.42	0.41-0.44
QoL-P.H **(T4)**	0.60	0.60-0.63	0.64	0.64-0.67	0.05	0.04-0.07	QoL-P.H **(T4** vs. **T0)**	1.26****	1.26–1.30	0.89***	0.89-0.93	0.28	0.28-0.30
QoL-S.R **(T0)**	0.54	0.54-0.57	0.99**	0.99-1.03	0.38	0.37-0.40							
QoL-S.R **(T3)**	0.01	0.01-0.03	0.29	0.28-0.32	0.34	0.33-0.37	QoL-S.R **(T3** vs. **T0)**	1.11***	1.11–1.14	0.67**	0.67-0.69	0.22	0.21–23
QoL-S.R **(T4)**	0.14	0.12-0.15	0.27	0.26-0.30	0.12	0.11-0.14	QoL-S.R **(T4** vs. **T3)**	1.22****	1.24–1.27	0.56*	0.55-0.57	0.01	0.01-0.02

A-MR: Ayahuasca-assisted Meaning Reconstruction group; MR group: Meaning Reconstruction group; NT group: No treatment group. T0: pre-treatment assessment; T3: post-treatment assessment; T4: 3 months follow-up assessment. TRIG: Texas Revised Inventory of Grief total score; TGI-SR: Traumatic Grief Inventory Self-Report total scores; PSGI-SF: Post-traumatic Growth Inventory-Short Form total score; QoL-P.H: Psychological Health domain of the World Health Organization Quality of Life (WHOQOL-BREF); QoL-S.R: Social Relationships domain of the World Health Organization Quality of Life (WHOQOL-BREF). *: Bonferroni p-adjusted value < 0.05; **: Bonferroni p-adjusted value ≤ 0.01; ***: Bonferroni p-adjusted value ≤ 0.001; ****: Bonferroni p-adjusted value ≤ 0.0001.

The within-group comparison performed to test the *Intervention x Time* interaction showed higher PTSG-SF scores for the A-MR group at both post-intervention time points [T0 vs. T3: *p* < .0001; Cohen’s *d* = 1.30; T0 vs. T4: *p* < .0001; Cohen’s *d* = 1.78]. The MR group showed higher PTSG-SF scores only at post-intervention assessment [T0 vs. T3: *p* = .016; Cohen’s *d* = 0.57]. However, the NT group did not show significant improvement in PTSG-SF at any post-intervention time point (T3 and T4: *ps* ≥ 0.999) (see Table 3 and Fig. 3a).

The RM-ANOVA performed to analyze the effect of treatment on the Psychological Health and Social Relationships domains of the WHOQOL-BREF showed *Intervention x Time* interaction (F_(4; 156)_ = 7.01; *p* < .0001; η*p*^*2*^ = 0.15; F_(3.47; 135.2)_ = 9.92; *p* < .0001; η*p*^*2*^ = 0.20; respectively). No main effect of the covariable “Months since death” was observed in Psychological Health (F_(2; 156)_ = 1.72; *p* = .182; η*p*^*2*^ = 0.02; F_(1; 78)_ = 1.29; *p* = .260; η*p*^*2*^ = 0.02), or in Social relationships domains (F_(1.73; 135.2)_ = 1.09; *p* = .330; η*p*^*2*^ = 0.01; F_(1; 78)_ = 0.571; *p* = .452; η*p*^*2*^ < 0.01).

The analysis conducted to assess the significant interaction of *Intervention x Time* for the Psychological Health domain of the WHOQOL-BREF did not show differences between groups at baseline (T0: *ps* ≥ 0.329). Differences were only observed between A-MR and MR groups at post-intervention [T3: *p* = .027; Cohen’s *d* = 0.66].

The within-group comparison did not show an improvement in the Psychological Health domain for the NT group at any post-intervention assessment (T3 and T4: *ps* ≥ 0.120). On the other hand, both the A-MR and MR groups showed improvements at post-intervention [(T3 vs. T0): A-MR: *p* < .0001; Cohen’s *d* = 1.18; MR: *p* = .008; Cohen’s *d* = 0.63] and at follow-up [ T4 vs. T0: A-MR: *p* < .0001; Cohen’s *d* = 1.26; MR: *p* = .0002; Cohen’s *d* = 0.89] (see Table 3 and Fig. 3b).

Finally, the between-groups comparison performed to analyze the interaction of *Intervention x Time* for the Social Relationships domain of the WHOQOL-BREF did not show differences at any post-treatment measurements (T3 and T4: *ps* ≥ 0.794).

However, a lower Social Relationships domain score was observed for the A-MR group compared with the NT group at baseline [T0: *p* = .002; Cohen’s *d* = 0.99]. The within-group comparison showed improvements in the Social Relationships domain at both post-intervention assessments for the A-MR group [T3 vs. T0: *p* < .0001; Cohen’s *d* = 1.11; T4 vs. T0: *p* < .0001; Cohen’s *d* = 1.22] and for the MR group [T3 vs. T0: *p* = .005; Cohen’s *d* = 0.67; T4 vs. T0: *p* = .019; Cohen’s *d* = 0.56]. No differences were found for the NT group at any post-treatment assessment (*ps* ≥ 0.807) (see Table 3**and** Fig. 3c).

## Discussion

In this open-label, three-arm, sequentially allocated study, we evaluated the potential benefits of an ayahuasca-assisted meaning reconstruction therapy for grief. Our findings provide preliminary support for the safe use of ayahuasca within this therapeutic protocol, suggesting a reduction in the severity of grief symptoms and PGD symptomatology, as well as the promotion of post-traumatic growth. The effects on these variables were superior to those observed with the therapeutic protocol alone, or the natural passing of time, and they remained stable for up to three months post-intervention. No serious adverse events were reported during the study. The incidence of minor adverse effects in the experimental group is aligned with prior research on ayahuasca use^32,33^.

The magnitude of grief severity reduction observed in this study was consistent with the outcomes from previous observational research on the impact of ayahuasca on grief^15^. Notably, in this study, less than half the number of ayahuasca sessions were needed to achieve twice the effect size, which was maintained at follow-up. This may be because, in this study, the ayahuasca sessions were part of a specific meaning-oriented therapeutic protocol to address grief, which has demonstrated promise in previous open trials^34–37^.

To our knowledge, only three studies have examined the efficacy of an early intervention on grief within the first year after the loss of a loved one^38–40^. Higher dropout rates, and lower intragroup effect sizes, were observed in such previous studies in comparison with the clinical outcomes reported here. In fact, the large effect size in the reduction of the severity of grief symptoms observed in the experimental group has not been found in any other intervention for PGD^26,41–44^. This may be attributable to the fact that one of the central challenges in grief is the meaningful integration of the loss into the bereaved individual’s life narrative^34,38^. Interventions that emphasize narrative retelling and meaning-making processes have demonstrated efficacy in facilitating this integration. Within this therapeutic framework, ayahuasca may act as a potent catalyst by eliciting emotionally salient autobiographical memories involving both the bereaved and the deceased, while fostering insights into the loss and its existential dimension, and facilitating identity reorganization^15–17^. These emergent insights can then be further explored, processed, and integrated during post-experience integration sessions through structured restorative narrative retelling. The dynamic interplay between the acute subjective effects induced by ayahuasca, the psychotherapeutic integration sessions, and the broader meaning reconstruction therapeutic sessions may thus generate a synergistic effect, enhancing the intervention’s capacity to address the complex psychological and emotional dimensions of grief.

Secondary outcomes also favored the Ayahuasca-assisted Meaning Reconstruction therapy group (A-MR) over control groups (MR and NT). Differences in post-traumatic growth were observed between the A-MR group and both control groups at post-intervention and over the NT group at follow-up assessments, with effect sizes ranging from medium to large. One of the psychological processes mediating post-traumatic growth when facing stressful events is the capacity to create meaning^45,46^. Several studies have proposed that psychedelics enhance the perception of meaning^47^which may function as a mediating mechanism underlying their therapeutic efficacy^48,49^. Our findings make it difficult to ascertain whether ayahuasca alone facilitates post-traumatic growth or if it results from the synergistic interaction with the integration and psychotherapeutic process. However, while an increase in post-traumatic growth is observed in the group that used ayahuasca (A-MR) at post-intervention assessment, the MR group needed a longer period to manifest this effect, only being observed at the follow-up assessment. The potential role of meaning-making as a mediator of primary and secondary outcomes will be analyzed in an upcoming publication^31^.

Multiple studies have observed a negative relationship between grief and quality of life among bereaved people^50–52^. However, only a few studies have found an acceptable effect size on quality of life after a therapeutic intervention^53,54^. In this study, a moderate-to-large intragroup effect size was observed in the domains of psychological quality of life and social relating in the A-MR and MR groups, which was not observed in the NT group. The larger intragroup effect size observed in the experimental group aligns with the outcomes of other studies on the effect of ayahuasca on the quality of life in bereaved people^15^as well as studies on psilocybin and its impact on the quality of life of patients with life-threatening cancer^55,56^ and depression^57^.

Finally, although previous studies have found a moderating effect of time since death on the efficacy of bereavement therapies^27^the covariate “Months since death” did not have a significant effect on grief severity improvement or PGD symptomatology in our sample. However, we observed an improvement in bereavement symptoms across the different assessment points for the 3 groups once the study began. Since at the baseline assessment not enough time had passed to determine whether the sample exhibited chronic grief, these changes may be due to the natural course of the bereavement process, as well as unidentified biological and psychosocial variables that promote adaptation. In addition, it is known that enrollment in a clinical trial can lead to symptom improvement regardless of the treatment group in which the participant is included. This phenomenon is often attributed to the impact of being closely monitored, as repeated assessment of symptoms can alter those symptoms^58^. This is partly connected to the Hawthorne effect, where a person’s behavior changes in response to the interest, care, or attention received by the study’s personnel^59^.

### Limitations

It is important to note further limitations in this study. Foremost among these is the issue of internal validity. The non-randomization of groups was a direct consequence of the COVID-19 pandemic, during which restrictions on gatherings and sanitary requirements to enter Spain were enforced. These circumstances led us to sequence group allocation to start with the control groups (MR and NT), in which onsite ayahuasca sessions were not part of the intervention protocol, so they could be postponed at the conclusion of the follow-up assessment without a specific date. Nonetheless, sequential allocation inherently lacks the methodological rigor of randomization in controlling for selection bias. Consequently, there exists a substantive risk that temporal confounders, such as differential elapsed time since the COVID-19 pandemic between groups, or procedural variations, including evolving staff expertise, may have systematically influenced outcomes. These factors compromise intergroup comparability and challenge the attribution of observed effects exclusively to the intervention under study. Furthermore, the study’s open-label design introduces a heightened potential for expectation bias, potentially impacting participant-reported outcomes, particularly given the reliance on self-report measures where subjective perception is paramount. Collectively, these design characteristics substantially limit the internal validity and generalizability of the findings, precluding definitive conclusions regarding the efficacy of the ayahuasca-assisted meaning reconstruction therapeutic protocol.

The second limitation involves external validity: the fact that three-quarters of the sample had used psychedelic substances in the past and were self-referred through the social media of psychedelic research institutions, may limit the generalizability of the outcomes. Expectancy effects could have influenced our results in the AM-R group, as prior use of psychedelics suggests that these participants may have had previous positive experiences.

The third limitation refers to the absence of standardized measures to capture the various dimensions of the negative effects associated with both psychological and pharmacological interventions, which leads to inconsistencies in the collected data and a potential underestimation of negative effects by participants. Furthermore, the lack of an independent rater to assess whether adverse events are attributable to the intervention necessitates a careful assessment and interpretation of participants’ reports of negative effects. This remains a critical issue in determining which patients may safely benefit from psychedelic therapy as a treatment alternative. Future studies would benefit from employing standardized assessment protocols that combine validated instruments with qualitative approaches to capture the complexity of potential adverse effects.

The fourth limitation has to do with the fact that no group received ayahuasca without therapeutic support, nor without preparation and integration sessions. Additionally, ayahuasca sessions were conducted in a group format, fostering the potential effect of mutual support and connection among bereaved participants. Moreover, the sessions took place in a natural setting, which may have influenced the participants’ sense of well-being. These non-pharmacological factors prevent the effect of ayahuasca alone from being properly isolated from the effect of these components. However, the aim of the study was not the development of ayahuasca as a stand-alone drug treatment, but rather to assess potential benefits associated with an early supportive intervention for bereaved people that integrates two models that have shown promising results in preliminary studies: the psychedelic-assisted therapy framework^60^ and meaning reconstruction approach to treat grief^35,61^.

Finally, the short follow-up period of three months restricts the ability to explore the long-term sustainability of the intervention and to assess the potential for symptom relapse.

## Conclusion

In conclusion, we aimed to examine the therapeutic changes associated with an ayahuasca-assisted meaning reconstruction therapy as an early resource to support bereaved people. We propose that the effects of ayahuasca may catalyze the psychotherapeutic work of meaning reconstruction, improving adaptation to loss. The magnitude and duration of the post-treatment reduction in the severity of grief and PGD symptoms, as well as the increase in post-traumatic growth, indicate the need for further research in this area. However, we would like to emphasize that consensual cooperation with Indigenous peoples and ayahuasca religious communities is essential to ensure the ethical and responsible use of ayahuasca in Western societies.

## Method

### Ethical considerations

The study was reviewed and approved by the Ethics Committee of the Hospital Universitari Germans Trias i Pujol (CEIC PI-19-188), Spain. The study was registered on clinicaltrials.gov on 2023-12-11 (NCT06150859). The trial protocol and statistical analysis plan have been previously published elsewhere^30^. The authors assert that all procedures contributing to this work comply with the ethical standards of the relevant national and institutional committees on human experimentation, and with the Helsinki Declaration of 1975. Informed consent was obtained from all participants.

### Trial design

This tree-arm sequentially allocated open-labeled study investigates the efficacy of ayahuasca-assisted meaning reconstruction therapy (A-MR) in decreasing grief symptoms severity and PGD symptoms in people who have lost a first-degree relative within the prior 12 months. Secondary outcomes involve post-traumatic growth, and the psychological health and social relationships domains of quality of life. Participants were sequentially allocated to the following treatment conditions, adhering to this predetermined order of assignment until the target sample size for each group was achieved: (1) a control group receiving Meaning Reconstruction Therapy (MR), (2) a control group receiving No Treatment (NT), and (3) an experimental group receiving Ayahuasca-assisted Meaning Reconstruction Therapy (A-MR). No measures were taken to achieve baseline equivalence between groups. Qualifying participants were assessed at baseline (T0), post-treatment (T3) and 3-month follow-up (T4). The subjective effects of ayahuasca were assessed in the experimental group (A-MR) on the following week after the ayahuasca administration (T1 and T2) (see Fig. 1). Results on the mediating potential of subjective affect and other mechanisms of psychological change will be analyzed in a future paper^31^. For ethical reasons, all participants who enrolled in either control group (MR or NT) and still showed severe grief after 3 months follow-up assessment (TRIG ≥ 40)^62^, were offered 2 voluntary ayahuasca sessions conducted over a single weekend, preceded by one online group preparation session and followed by two online ayahuasca integration sessions, conducted over the two weeks following the ayahuasca session^31^.

### Sample size calculation

The sample size of 69 participants was calculated using the pwrss R package (version 0.3.158)^63^with a power of 0.90 and an alpha of 0.05. To account for a 20% drop-out rate, the final sample size was adjusted to 84. The sample size estimation was based on the effect size and the TRIG measurement correlation of 0.55, from a study by González et al.^15^.

### Participants

Participants were recruited from October 10, 2021, to April 15, 2024, advertising in the social media accounts of several international institutions related to psychedelic research. All participants met the following inclusion criteria: (1) aged 18 to 65, (2) having experienced the loss of a first-degree relative (3) within the past 12 months, and (4) with a Texas Revised Inventory of Grief score over 39 (TRIG ≥ 40). Exclusion criteria involved: (1) diagnosis of a medically significant health condition; (2) previous or current history of a psychotic disorder (Axis I- DSM-IV-TR); (3) pregnant or breastfeeding women; (4) hypertension (systolic blood pressure (SBP) > 140 mmHg, diastolic blood pressure (DBP) > 90 mmHg, heart rate (HR) > 100 bpm); (5) substance use disorder, excluding nicotine; (6) alcohol consumption exceeding 40 g/day; (7) concurrent psychological or pharmacological treatment for grief.

All participants provided written informed consent prior to enrollment in the study. Informed consent was obtained by providing participants with clear and comprehensive information about the study’s purpose, procedures, potential risks, and benefits. Risk disclosure included a detailed explanation of possible adverse effects or discomforts associated with the study. They also had the opportunity to ask questions during an online call prior to providing consent. Participants who gave written informed consent were screened for study eligibility and examined for psychiatric conditions by the principal investigator. Blood pressure was assessed by the medical doctor of the study on-site, before each ayahuasca session. Participant safety was monitored throughout the dosing session by trained staff to ensure their well-being.

### Outcome measures

Severity of grief was assessed with the Texas Revised Inventory of Grief (TRIG)^64^and prolonged grief disorder symptoms following the DSM-5-TR^65^ were assessed using Traumatic Grief Inventory-Self Report (TGI-SR)^66^. Secondary measures included posttraumatic growth, assessed with the Posttraumatic Growth Inventory-Short Form (PSGI-SF)^67^ as well as Psychological Health and Social Relationships domains of quality of life, measured using the corresponding scales of the World Health Organization Quality of Life-BREF instrument (WHOQOL -BREF)^68^. Adverse events were collected 24 h after ingestion by a physician using the excitement component of the Positive and Negative Syndrome Scale (PANSS-EC)^69^. Moreover, participants answered the following questions at post-assessment: (1) “Have you experienced any negative effects from the psychotherapeutic sessions?”; (2) “Did you experience any negative effects during the ayahuasca session?”; (3) “Did you experience any negative effects in the week following the ayahuasca session?”. Responses were categorized as: (1) No, (2) Uncertain, or (3) Yes. No standardized instruments were used to assess adverse events by the participants. Two satisfaction-related questions were added at post-assessment: (1) “To what extent are you satisfied with the therapeutic support provided during the psychotherapy sessions?”; (2) “To what extent are you satisfied with the ayahuasca sessions?”. Responses were rated using a 5-point Likert-type scale (from 1 = “Very dissatisfied” to 5 = “Very satisfied”). These questions were followed by an open-ended prompt encouraging participants to provide further information about their responses. Responses are presented in the supplementary material to complement and illustrate the quantitative data. A qualitative analysis of these narratives will be undertaken in a subsequent sub-study.

### Interventions

#### Components of the support program

The therapeutic protocol, part of both the A-MR and the MR groups, is grounded in a meaning reconstruction approach^38,70^. This model focuses on the crucial role of meaning-making in grief recovery, and posttraumatic growth, after the death of a loved one^71,72^. The intervention is structured around a three-phase framework aimed at supporting bereaved individuals in overcoming challenges related to sense-making and meaning-making around the loss, revising and redefining their attachment to the deceased, and reorganizing their sense of identity in response to the bereavement experience. Participants were offered a total of 9 online 50-minute sessions, on a weekly basis, conducted by a team of 4 clinical psychologists. Therapeutic sessions were conducted online through the Zoom for Healthcare platform, in compliance with HIPAA guidelines. Phase 1 (Session 2–3) addressed the “event story” of the loss, in order to process traumatic or stressful memories; Phase 2 (Session 4–6) focused on the “back story” of the relationship with the deceased, to restore attachment security through reconstructing a bond with the deceased (if appropriate) and to work through potential unfinished business; Phase 3 (Session 7–8) focused on the “personal story” of the participant’s self, to reorganize their identity as a survivor (see^31,73^for a case report and a detailed therapeutic protocol respectively).

### Ayahuasca sessions and integration

Ayahuasca sessions for the A-MR group were carried out at the IREHOM center in Barcelona, Spain. Ayahuasca sessions were conducted in groups of a maximum of 8 participants, supported by a minimum of 4 facilitators, after Phases 1 and 2 of the therapeutic protocol. Participants were encouraged to lie on a couch and listen to a standardized playlist of music on a speaker. An online individual psychedelic integration session was conducted after each ayahuasca session, during the following week, in alignment with the non-directive principles of restorative retelling^74,75^. Details of the procedure of the ayahuasca session have been previously published^31^.

### Ayahuasca analyses and dosage

A ten-liter batch of ayahuasca was donated by CEFLURIS (idaris.com.br/en), prepared by boiling the stems of *Banisteriopsis caapi* and the leaves of *Psychotria viridis*. Analyses were carried out by Energy Control (energycontrol-international.org) using liquid chromatography- mass spectrometry (LC–MS). The Ayahuasca brew used contained 0.6 mg/ml of N, N-dimethyltryptamine (DMT), 1.1 mg/ml of tetrahydroharmine, 0.03 mg/ml of harmaline, and 4.2 mg/ml of harmine and 0.01 mg/ml of N-methyltryptamine (NMT). A medium dose of ayahuasca (0.75 mg DMT/kg)^76^ was orally consumed in two different intakes (0.30 mg DMT/kg as an initial dose and 0.45 mg DMT/kg after 30 min).

### Safety measures

Blood pressure and heart rate were assessed one hour before each ayahuasca session and 6 h after administration. Medication was available to treat headaches, hypertension, severe anxiety, or psychotic symptoms. Participants remained in the center for a 24-hour period, under medical vigilance.

### Statistical analysis

Statistical analyses were conducted following the best practice recommendations for clinical trials analysis^77,78^. As this is an exploratory study, a completer analysis strategy was used^79^. Before the study analysis, data were explored for outliers, missing variables and drop-out. To test the possibility that missing data could be considered as Missing Completely at Random (MCAR), Little’s MCAR test and logistic regression analyses were used. Missing data were imputed simultaneously using Multivariate Imputation by Chained Eq.^80^. All the study variables were included for missing values imputation using the predictive mean matching methods and 5 multiple imputations.

Descriptive statistics were reported for the total sample and for the 3 groups studied: Meaning Reconstruction Therapy (MR), No Treatment (NT) and Ayahuasca-assisted Meaning Reconstruction Therapy (A-MR). Between groups differences at pre-treatment were analyzed using ANOVAs, Kruskal-Wallis, Chi-square or Fisher’s exact test for the continuous, ordered and nominal variables, respectively. Drop-out participants for each group were reported and between drop-out and completers differences were tested using one-sample Student’s *t*-test.

The effects of treatment on the primary (TRIG and TGI-SR) and secondary outcomes (PSGI-SF, WHOQOL-BRIEF Psychosocial Health and Social Relationships scales) were studied using repeated measures analysis of covariance (RM-ANCOVA). The RM-ANCOVA was used because time since death was included as a covariate in the analyses to test the effect of this variable on outcomes. Prior to performing the RM-ANCOVAs, applicability criteria for the test were analyzed. The RM-ANCOVAs included 2 fixed factors with 3 levels each (Time: pre, post-treatment and follow-up; Treatment: NT, MR and A-MR) and months since death as a covariate. Moreover, pre-treatment measures were also included as covariates in the RM-ANCOVAs, if significant differences at pre-treatment were observed. Greenhouse-Geisser correction was used when Mauchly’s test for sphericity was significant with epsilon < 0.60, while Huynh-Feldt correction was used with epsilon values ≥ .60^81^. The treatment effect was studied analyzing the Treatment x Time interaction. The effect of months since death on the participants’ response was analyzed when a significant interaction with Time was observed. Significant interactions were analyzed by paired and unpaired Student’s *t*-tests. Bonferroni corrections were applied to all analyses and only adjusted p values are reported in the Results section. Partial eta-square and Cohen’s d effect sizes were reported for the interaction effects and for between and within groups comparisons, respectively. Differences between the ayahuasca group participants who attended to one or two ayahuasca sessions was tested using nonparametric independent samples test. Confidence intervals for Cohen’s d was estimated with a 95% confidence level and 5000 bootstrap samples.

The statistical analyses were performed using R version 4.2.3^82^. The following R packages were used for data exploration, description, and preprocessing if necessary: tidyverse^83^car^84^moments version 0.14.1^85^, and nortest version 1.0-4^86^. The RM ANOVAs and ANCOVAs were performed using the rstatix package version 0.7.2^87^ and/or car package version^88^. Finally, missing variables were explored by naniar package^84^ and missing data was imputed by MICE package version 3.16.0^89^.

## Supplementary Information

Below is the link to the electronic supplementary material.

Supplementary Material 1

## Data Availability

Data from this study are available from the corresponding author upon reasonable request, subject to ethical and confidentiality considerations.
