# Supplementary material for: Ayahuasca-assisted meaning reconstruction therapy as an early resource for bereavement: a non-randomized clinical trial
Source: Sci Rep. 2025 Sep 1;15:32158. doi: 10.1038/s41598-025-13251-5 (PMC12402194; doi:10.1038/s41598-025-13251-5)
Supplement: Supplementary file 1 — Supplementary Material 1 [file 41598_2025_13251_MOESM1_ESM.docx]

**Appendix. 1**

Adverse effects related to ayahuasca administration (A-MR group: n = 27)

**(1) “Have you experienced any negative effects from the psychotherapeutic sessions?”**

| No [n = 53 (96.36)] | Uncertain [n = 1 (1.82%)] | Yes [n =1 (1.82%)] |
| --- | --- | --- |

**(2) “Did you experience any negative effects during the ayahuasca session?”**

| No [n= 11 (40.74%)] | Uncertain [n= 8 (29.63)] | Yes [n = 8(29.63)] |
| --- | --- | --- |

**(3) “Did you experience any negative effects in the week following the ayahuasca session?”**

| No [n= 24 (88.89%)] | Yes [n = 3 (11.11%)] |
| --- | --- |

**Open-ended information.**

*Text translated from Spanish into English

1. A very intense headache, concentrated in the forehead area during the ceremony that lasted for an hour or two, and then the next day with less intensity and time.
2. A lot of somatic issues during the ceremonies such as muscle pain, headache, diarrhoea, and minor sickness for several days.
3. I don't know if I should consider it an adverse effect because it helped me open up to the experience when I was able to vomit*.
4. I passed out, and the effects wore off*.
5. One session was more difficult for me because I felt some anxiety when I connected with the pain of my mother's death. My lower back hurt, and I purged a lot. I have a lot of respect for the severity with which the trip was presented to me*.
6. I felt the urge to go to the bathroom, but I couldn’t. Had almost no strength to move*.
7. Every time I heard someone throwing up, I felt nauseous. In the end, I threw up and felt at peace*.
8. In the second session, I felt a deep anguish that I thought was overtaking me, and pain in my heart that passed when I was able to breathe better outside of the room. My temples were aching from the amount of snot and tears I had been trying to absorb*.
9. I vomited spectacularly. Good thing I had the bucket next to me!*
10. I spent a good part of the session in the bathroom, and even though I hadn't eaten anything or drunk anything for several hours, I was vomiting a lot of liquid that I don't know where it came from*.
11. I felt a little uncomfortable, restless, and nauseous*.
12. Some moments were so intense that I had to take deep breaths to deal with it and try not to vomit*.
13. I did not like the experience. At first, I was relaxed, but then the intensity began. I started to feel nausea, discomfort, anguish, a sense of losing control, until I lost consciousness. I had to take an ibuprofen at night because I had a headache*.
14. I think some of the sounds [I was hearing] made me sick to my stomach and upset me. At times, I felt very irritated*.
15. The first few weeks after the intake, I was quite distracted, as if I had been left in limbo*.
16. The experience became too intense. When I thought I was feeling better, the nausea would return after a while. I was very sensitized to any sound and movements in the room*.
17. The [ayahuasca] shot was revealing. I felt like throwing up, especially when I had some realizations about my mother and, thus, also about myself*.
18. Sometimes, the nausea prevented me from concentrating on what I had to work on*.
19. A little bit of stomach discomfort and movement of the viscera*.
20. Just normal purging, but I don't take it as an adverse effect, but as a detoxification*.

**Appendix. 2**

**(4) “To what extent are you satisfied with the therapeutic support provided during the psychotherapy sessions?**

**(5) “To what extent are you satisfied with the ayahuasca sessions?**

|  | **Therapeutic sessions** | |  | **Ayahuasca sessions** | |
| --- | --- | --- | --- | --- | --- |
|  | **n= 55** | **%** |  | **n = 27** | **%** |
| Very satisfied | 41 | 74.5 |  | 22 | 81.5 |
| Somewhat satisfied | 12 | 21.8 |  | 3 | 11.1 |
| Neither satisfied nor dissatisfied | 0 | 0 |  | 0 | 0 |
| Somewhat dissatisfied | 1 | 1.8 |  | 1 | 3.7 |
| Very dissatisfied | 1 | 1.8 |  | 1 | 3.7 |

Level of satisfaction with the therapeutic sessions (A-MR and MR group) and the ayahuasca sessions (A-MR group).

**Open-ended information.**

*Text translated from Spanish into English

The personal names of psychologists have been replaced by “the therapist.”

1. The therapist has been very helpful for me and she has understood where I am. I have enjoyed the sessions with her.
2. It was very helpful to "let it all out" without fear of judgment. To have someone to share some of my loved one’s "secrets" with that I could not share with others for fear they would have a different type of opinion about him. It helped me to sort things out in my own mind. Things are still rather muddled, but if my brain can be compared to a messy room, at least I have taken a step or two to start sorting through the mess.
3. Having little experience with psychotherapy I wasn't sure what to expect but found myself looking forward to the sessions with the therapist. He seems like a good, understanding guy and I feel he helped me to begin the healing process.
4. I was terrified at the start of the process and doubted that I would complete the therapy.
5. I felt that during my sessions I was free to express my feelings safely and openly while given subtle but clear direction on what part of my emotions to explore. My therapist was a very calming presence and was able to expand my thoughts about events and my feelings.
6. I feel that therapy has helped me a lot, now I don't feel constantly depressed like I did before. It's just more occasional moments.
7. In one way I felt that it took 2 or 3 sessions to trust my therapist and so I did not get as deep into certain things that I wanted. Although I realise that this was only related to grief, the death of a child raises many questions about your own upbringing and the relationship with your family and I felt there wasn’t time to explore this.
8. The therapist was very empathetic. He was always very understanding and supportive. He made a couple of helpful suggestions which I have found useful. I don't think he could have helped me much more as the enormity of my tragedy cannot really be overcome by talking/therapy - suicide is too big for that. But it was useful and I am glad I went through with it.
9. The psychotherapeutic sessions have been a great resource, a channel that allows me to focus on the grieving process. The therapist has been exceptional, and the exercises provided by her, have been a great way for me to think about all the grieving process, allowing me to connect with past memories.
10. I am very happy with my therapy sessions. I found them very helpful and feel I would benefit therapy like this in the future.
11. I found the psychotherapy sessions very helpful. I felt very safe in the space created and it was very cathartic to share with her my experience of my Dad passing away. I have learnt some great tools to reconnect with my Dad, to visit him in my mind and to reconnect with myself after feeling like I was lost. The therapist helped me remember important lessons I learnt in caring for my Dad at the end of his life, which has helped me translate these into certain parts of my life now so I see them in a more positive way.
12. I found the psychotherapy to be extreme helpful. It really helped me to think about the passage of time, changing emotions, and not to put pressure on myself not to express my sadness and grief. I always feel the need to be strong for others and now I feel better about showing others how I feel.
13. There were sessions where I felt like I passed a huge obstacle. But as I normally introspect, some sessions were not as effective for me. It eased a lot of this anguish and terror and would sometimes come in the right time when a pang of grief hits me.
14. I never thought I that I could tell someone all my emotions and feelings and the different encounters that I have had with open extreme confidence and that has no judgment or awkwardness to my pain. I thought there was nowhere to turn, but these sessions and the person who hears me, is the best. I’m very grateful that this is helping me understand this unimaginable life lesson that is unbearable most of the time and leaves you in between dimensions of spinning darkness.
15. Therapy was a huge blessing, dedicating time to speak about my brother, having a space to process all the emotions and understanding the layers of grief has helped me heal in numerous ways.
16. The therapist was very kind, empathetic and patient. On the other hand I feel we barely touched the core of my problems, since we didn't have enough sessions and space for that.
17. I am very pleased with the psychotherapy sessions. I think they helped me talk about my grief and gave me an outlet to express my feelings.
18. It helped me in the grief process on a deeper level.
19. Upfront I didn't know what to expect of the psychotherapeutic sessions. The way we, my wife and sons, were dealing and handling our grief felt OK to me. I didn't feel the need for psychotherapy. Nevertheless, I experienced the conversations with the therapist as helpful. Helpful as it provided a way to talk about my son, which helps in capturing memories and not to forget. Speaking about my son is always very emotional, but that is OK. It scares me more when I think of him without the pain and sadness. I embrace the grief, as if it brings me closer to him.
20. I had a very supportive environment, my psychotherapist was great, she made me feel very comfortable when talking to her, she has truly facilitated in changing the way I see the world. The integration was great and really necessary. I am so excited to live my life, ayahuasca and psychotherapy have been truly a blessing, I never thought I would get such clarity regarding patterns that are detrimental to me. Thanks so much for everything.
21. The therapy was a key element for me. It made me deal with some of the traumatic experiences I had with my son’s sickness and death in a direct way which was what I needed as before the study started I would have overwhelming feelings of sadness randomly and I wouldn't know how to cope with these feelings. The therapy brought up many unconscious feelings to my conscious mind and allowed me to deal with them. The therapist was a great support for me and I feel that she really understood me.
22. I felt very comfortable and listened to, although I perceived the rigidity of the therapeutic protocol, given that a grieving process is a network of interrelated relationships*.
23. Excellent work of support, focusing on the positive aspects in relation to the grief of the loved one, and reflecting on issues that you do not give enough space*.
24. I feel that I am in total confidence, a space where I can open up and express what I feel in that moment of truth. I have felt the anguish subside and transform as the sessions progressed*.
25. It has helped me to heal many aspects of my past life with my son, and to remember him with affection*.
26. The therapist has helped me to accept that I did everything I could under the circumstances and that there is no one responsible for a suicide*.
27. It has helped me a lot to have a therapeutic accompaniment during these months, it has been key to be able to integrate the event and move forward*.
28. He has accompanied me without pressuring me at any time and I have been able to advance in my understanding of what has happened at my own pace. I am trying to form my own sincere opinion about the continuity of existence after death. The therapist has accompanied me and in a way has guided me, but at no time has she tried to influence my beliefs. She helps me to follow my path to arrive at whatever my personal conviction is (I am still working on it). It has made me think a lot, especially in the times between sessions, as a result of what was discussed in those sessions*.
29. Empathic support, with a good connection, close, helpful, revealing, a smooth and harmonious process, without jumps or abruptness even though I had to reschedule some sessions*.
30. I felt very accepted and free to express myself spontaneously*.
31. There was a lot of connection with my therapist, she made me reflect on aspects of myself and my life that I had not paid attention to, I looked forward to each session, it was also very good how she made me see the accomplishments that I have been achieving little by little to be better*.
32. It has helped me to integrate everything that has been happening to me and has helped me to talk about my loved one as well as what kind of relationships I have now with the people around me*.
33. The accompaniment has been fundamental, not only because it has been kind and respectful, but also because it has offered me really deep and extremely useful insights; it has shown me many new possibilities and ways of doing and being in my life in general. And it has helped me a lot to integrate the experiences with Ayahuasca, allowing me to ground messages and great understandings applicable to my daily life*.
34. It has been very easy for me to open up to her, something that had never happened to me before. Her rhythm management and her proposals made me feel that we were doing tangible, real and profitable work at all times. No filler. Her ability to listen and to take me to memories and situations, her empathy, her *compassion*. I feel very fortunate to have had her as a therapist during this process. I have always found her considerations or assessments to be intelligent, gentle, accurate*.
35. Her help with integration has been invaluable. Had it not been for her, 80% or 90% of the messages received in the ceremonies, especially in the second one, which was much more confusing, would have remained unintegrated and things that for me are now very important and transcendent would have remained mere anecdotes without much meaning*.
36. The work of the whole team in the ayahuasca sessions, as well as the therapist in particular in the therapy sessions has been deeply healing. I loved it :)*.
37. The combination of therapy and ayahuasca intakes, plus the careful set and setting as well as the depth of the commitment from where the proposal is made and finally the all-female team as a whole, all add up to the excellence of the experience. Totally transformative*.
38. It has helped me a lot to process and integrate my loss and has been very professional and humane*.
39. I felt understood*.
40. It was very good to be able to explain my doubts and concerns and to reflect on different points of view*.
41. Beautiful experience from the human point of view, the medicine was what I didn't like. It was the most dreadful headache I have ever experienced in my life *.
42. Excellent therapist, great person and very professional, we have done a great job together*.
43. I notice a profound change. There is still a long way to go because doors have been opened to the unknown but in relation to the mourning, the anger has been extinguished, the desire for reparation is becoming more and more pregnant. Things are slowly falling into place. Thanks to all of you*.
44. It has given me the possibility to contemplate new ways through which I could reduce and even eliminate the vital anguish and disorientation in me*.
45. Empathy, professionalism and a lot of good listening*.
46. I felt very well accompanied. My therapist was empathic and her impartial, curious and non-judgmental presence allowed me to open up in my process*.
47. I felt very accompanied, understood and respected by the therapist, some of her words have stayed with me and have allowed me to go a step further on my way to find myself and my life*.

**Ayahuasca ceremonies:**

1. The ayahuasca sessions were incredible, especially the second session. They changed my outlook on life and the death of my father. I am very glad I participated and I’m not sure if the therapy alone would have had the same effect.
2. The experience to gather with other people with the same destiny was a healing process. The second ayahuasca made me understand life. Everyone attending are loving people.
3. I went into the first session with sky high expectations or hopes to connect with my son. That didn't happen which in the first instance felt as a huge disappointment. On the other hand, I quickly realized the intense love I experienced, which even now when writing this down makes me very emotional. If I have to summarize my ayahuasca sessions it is precisely that: experiencing deep intense love. The emotions are enhanced by the surrounding, music and by the incredible staff. The care the staff surrounded us with was so intense that it stays with me and has changed me (I hope). After the second session I felt sad that it is over. I am extremely grateful for this experience.
4. The team of facilitators was great, supportive, and caring to the moon and back.
5. I never really knew that Ayahuasca could cause such an impact on people’s lives. The amount of compassion, clarity and love that has come back to me in these past two months has been a miracle. I feel integration is very important, but ayahuasca itself I can only say, is necessary for humans.
6. This medicine will be very important in my life, I had never felt so happy, and it still brings a smile to my face to know how lucky I have been to have this opportunity and experience. Again, thank you so much. I hope people like myself and who are suffering can have more access to this medicine, and I truly hope I can promote and work with it in the future to try and help others in need. Thanks again.
7. The first ayahuasca ceremony was an experience that I will never forget. I vividly remember the visions and intense emotions I had. This session unlocked a new part of me. The few weeks after that session was the best I had felt in a long while. The combination of the therapy with ayahuasca was a key element. The therapy brought the feelings I needed to deal with to the surface, and the ayahuasca helped me properly deal with those feelings.
8. It helped me reconnect with my mother, which, in turn, allowed me to find calm and release feelings of anger, anxiety, and guilt. Of course, it hasn’t erased my sadness, but it has helped me process it in a much healthier way*.
9. Both ceremonies were flawless—both professionally and spiritually. We received clear, simple safety protocols, and the setting was comfortable and secure. The food and lodging were excellent, and the generosity, respect, and care from the entire team created a sacred and nurturing atmosphere. It was an incredibly fulfilling and integrative experience*.
10. The sessions felt luxurious in every sense. The environment was ideal for healing, and every detail was of the highest quality. From the pre-ceremony rituals to the main ceremony and the post-ceremony care, everything was handled with extraordinary delicacy, care, and love. This allowed me to approach the experience with a sense of peace and focus. While the sessions had their challenges, I always felt supported and safe, which enabled me to fully surrender to the process. The positive impact of this support cannot be overstated*.
11. Personally, I would have appreciated a small spiritual ritual before the ceremony, such as asking for protection from God or nature, or offering gratitude at the end, but this is a minor detail. The overall event design, the preparation, the atmosphere, the location, the music, and the integration circles were flawless. For me, the presence of the Santo Daime group was invaluable—their serenity and sense of security were indescribable*.
12. Ayahuasca revealed wounds I had long buried. It made me realize how these wounds have shaped me and helped me develop a deeper understanding of myself. This has been an incredibly introspective journey*.
13. In my view, ayahuasca is an excellent companion in the grieving process, especially in this dosage. It allowed me to stay connected to my loved ones throughout my life, to heal, and to revisit past experiences in a way that transformed my grief. The preparatory work surrounding grief was also essential*.
14. I believe additional sessions could be beneficial. While the work we have done so far feels sufficient for now, I see potential for further growth in the future*.
15. This experience has been completely new for me—one that allowed me to express long-suppressed emotions. It has also helped me uncover realizations that might have otherwise remained hidden*.
16. I deeply value the people who supported me throughout this journey. Their warmth and presence were invaluable. These ceremonies represent a significant step on my path, and I acknowledge this milestone with gratitude*.
17. On a different note, while my first experience was extremely positive, the second session didn’t quite match the intensity of the first. Despite that, I still found it valuable and unique. However, I did not notice a substantial transformation in myself after the second session*.
18. I am forever grateful to the medical team. Their exceptional care and support made me feel completely safe and looked after at all times. Thank you so much!*
19. I feel that I have fully accepted my father’s passing. I have connected with immense beauty and love, and I now love and support myself more than ever*.
20. I sense a profound transformation within me. There is still a long road ahead, and I have opened doors to the unknown. But in terms of my grief, the anger has dissolved. The desire for reconciliation is growing stronger, and things are slowly beginning to settle*.
21. Ayahuasca allowed me to connect with my father, to say goodbye, and to truly understand*.
22. It has given me a new perspective on my life—where I come from and where I am going*.
23. I am deeply satisfied with the ceremony, as it provided me with a profound understanding of my life journey. However, it was also an intense and challenging process, one that has deepened my respect for this medicine*.
24. The total confidence I had in the facilitator and his team allowed me to embark on this journey without being paralyzed by the initial fear I felt. The choice of location was perfect, and I appreciated the opportunity to take ayahuasca during daylight hours. While taking two doses in succession initially seemed overwhelming, my experience from the first session allowed me to go even deeper in the second*.
25. Ayahuasca has given me more perspective on my life—where I come from and where I am headed*.
